# Predictors of Loneliness in Parkinson's Disease and Craniocervical Dystonia

**DOI:** 10.1002/mdc3.70098

**Published:** 2025-04-29

**Authors:** Suzette Shahmoon, Dejan Georgiev, Paul Jarman, Kailash Bhatia, Patricia Limousin, Marjan Jahanshahi

**Affiliations:** ^1^ Department of Clinical and Movement Neurosciences UCL Queen Square Institute of Neurology London UK; ^2^ Department of Neurology University Medical Centre Ljubljana Ljubljana Slovenia; ^3^ Faculty of Computer and Information Sciences University of Ljubljana Ljubljana Slovenia

**Keywords:** loneliness, Parkinson's disease, focal/segmental craniocervical dystonia, optimism and stigma

## Abstract

**Background:**

Loneliness is a state in which an individual feels socially isolated due to deficiencies in the quantity or quality of social relationships and interaction. To date very little is known about loneliness in Parkinson's disease (PD) and focal/segmental craniocervical dystonia (FSCD).

**Objectives:**

To explore whether level of loneliness is disease‐specific by comparing PD, FCSD and healthy controls (HCs), and to define predictors of loneliness in both PD and FSCD.

**Methods:**

Eighty‐two people with PD, 63 people with FSCD and 50 HC were surveyed. The UCLA Loneliness Scale was used to assess loneliness. Various non‐motor symptoms, psychosocial variables and measures of subjective well‐being were assessed and used as potential predictors of loneliness.

**Results:**

There was no significant difference in reported levels of loneliness between people with PD and matched HCs, and between people with PD and people with FSCD, but people with FSCD reported higher levels of loneliness than HCs (*p* = 0.018). Perceived stigma predicted loneliness in both disease groups (*p* < 0.001). Additionally, non‐motor symptoms (*p* = 0.006), lack of optimism (*p* = 0.015) and practical social support (*p* = 0.006) predicted loneliness in people with PD. Patients with PD and FSCD with higher perceived stigma levels felt lonelier (*p* < 0.001), as did female patients with PD (*p* = 0.004), younger patients with FSCD (*p* = 0.007) and older patients with PD (*p* = 0.023).

**Conclusions:**

We identified important predictors of loneliness in PD and FSCD. The identified age‐ and gender‐specific differences in loneliness in people with PD and FSCD contribute to our better understanding of this complex and not yet fully understood concept.

Loneliness is a state in which an individual feels socially isolated^1^ due to deficiencies in the quantity or quality of social relationships and interaction.^2^ This deficiency is experienced in a negative, distressing manner, and can encompass feelings of isolation even when in the company of others.^3^ There are individual differences with regards to how much good quality social interaction one needs, and this could be explained in part by personality. There has been surprisingly little research on personality and loneliness. However, the few studies that exist have shown extraversion to be consistently negatively associated with loneliness.^4^, ^5^ Extraverts have an under‐aroused nervous system and tend to seek out social stimulation, whereas introverts have over‐aroused systems and need more calm and quiet.^6^ This therefore explains why extraversion predicts greater participation in social activity^7^ and more perceived support from peers^8^ and its negative association with loneliness.^4^, ^5^ Optimism predicts higher psychological well‐being and quality of life.^9^ It is important to remain realistic with regards to what can and cannot be achieved while living with an illness.^10^

When loneliness is not addressed it can have serious consequences on the individual's cognition, emotions, behavior and health.^11^ A meta‐analysis that assessed the risk that loneliness poses to health, found that actual and perceived social isolation both increase the risk for early mortality.^12^ These findings were consistent across age, gender and world regions and were influenced by initial health status. What this review highlighted as well as the implications for health, is the subjectivity of the experience of loneliness. It is the individual's perceived lack of sufficient and good quality social interaction and support which can affect self‐care and mortality.^13^, ^14^ In 2021–2022 the British government conducted “The community life survey 2021/2022” which assessed well‐being and loneliness of over 17,000 participants of different ethnicity, socio‐economic and health status. The results showed that people with disability were significantly more likely to report loneliness, low social support and social isolation than non‐disabled individuals.^15^

Parkinson's disease (PD) is a disabling, neurodegenerative disorder, characterized by primary symptoms of tremor, bradykinesia and rigidity^16^ each of which can impact on the social activity of people with PD.^17^, ^18^ Yet the physical aspects are not the only factors which cause people living with PD to feel isolated. Facial masking in PD leads to fewer facial expressions than in healthy controls (HC).^19^ Facial masking makes it harder to express emotions, which can impact the ability to connect with others.^20^ Hypophonia and interrupted speech patterns can also cause people with PD to struggle to effectively communicate and interact with others. Dysarthria, which causes abnormal speech rhythm, a harsh voice, inappropriate pauses, and prosodic loss can also affect communication.^21^ These very visible and audible symptoms can affect other people's reaction to the person with PD and the person's own sense of self, resulting in them discrediting or devaluing themselves. Both of these can lead to the experience of stigma.^20^, ^22^ Stigma is the negative evaluation of a person as tainted or discredited on the basis of attributes, such as disease, including neurological and mental disorders, ethnicity, drug misuse or physical disability, and can be internal, perceived stigma and external, enacted stigma.^23^ Perceived stigma is a highly subjective phenomenon. Levels of perceived stigma experienced are very personal to the individual and often it is an amalgamation of many factors which contribute to stigma: gender, age, age of onset, culture, severity of motor symptoms and medication side‐effects such as dyskinesias.^24^ Both, perceived and enacted stigma have negative social, political, economic and psychological consequences for the stigmatized, and they may become insecure on how others will accept them and become self‐conscious about the impression they make on others.^23^

When socializing, people with PD can struggle due to their motor and non‐motor symptoms and dyskinesias, that may make them feel that they fail to meet social norms; which may in turn influence their self‐esteem, and increase their perceived stigma and in the longer term affect their ability to sustain relationships with family, friends and colleagues leaving them feeling isolated and lonely.^25^ The non‐motor symptoms of PD, such as depression, anxiety and cognitive impairment have also been associated with increased loneliness.^26^ A longitudinal study which assessed the effects of lockdown during the COVID‐19 pandemic showed an improvement in the non‐motor symptoms of anhedonia and apathy, due to the increased social support and presence of family during this period.^27^ Apathy is very common in PD and is often associated with loneliness as it can lead to withdrawal from social interactions, which in turn promotes feelings of loneliness.^28^ Hope is another important determinant of loneliness and can be instrumental in alleviating loneliness and promoting social support, which is also very important in determining loneliness.^28^ Social interactions provide emotional resources that help individuals cope with life's challenges. Conversely, a lack of social support can exacerbate feelings of loneliness and lead to negative effects on mental health.^29^ Happiness and loneliness are very often seen as exclusive categories. Loneliness can significantly detract from overall happiness, leading to feelings of sadness, anxiety, and depression.^29^

While it is important to understand if people with PD are more prone to loneliness compared to HCs it can also be useful to compare loneliness levels to those of people with another movement disorder. Dystonia refers to a group of heterogenous, movement disorders characterized by muscle spasms, uncontrollable repetitive movements, and contorted postures^30^ which can cause pain and reduce people's ability to work^31^ and socialize.^32^ Loneliness can be a significant issue in dystonia due to many different factors. The physical symptoms of dystonia can make social interactions difficult and lead to social isolation.^33^ Furthermore, like PD, anxiety and depression, which are also very common in dystonia, can exacerbate loneliness.^34^ Other factors, including happiness, hope, chronic pain and discomfort might also be related to loneliness in dystonia.^33^, ^34^ A survey of perceived stigma in people with cervical dystonia reported 93% of respondents feeling sometimes or definitely self‐conscious, 91% felt sometimes or definitely odd and different, 86% felt unattractive, 81% felt apologetic, 77% tended to avoid social contacts, and 45% felt being avoided by other people.^35^ Similar experiences of perceived stigma were also documented in a sample with another form of dystonia, spasmodic dysphonia.^36^ Such experiences of perceived stigma together with depression which is common in dystonia^37^, ^38^ can lead people with dystonia to report feeling lonely.^39^ Similarly, more than 50% of the patients with PD try to hide their diagnosis, or mask some of the symptoms or even avoid appearing in public.^40^ Making a diagnosis of PD or dystonia is a necessary step towards appropriate treatment of these disorders, however, this categorization is directly associated with stigmatization, for it is embedded in the social, cultural and political environment of the subjects suffering from them.^40^

The main aim of our study was to investigate the potential impact of motor and non‐motor symptoms of PD and FSCD on loneliness compared to healthy controls and the mediating role of apathy, depression, hope, happiness, perceived stigma, self‐esteem and personality. We hypothesized that both patients with PD and FSCD would be lonelier compared to healthy controls. We examined the potential contributions of anxiety, depression, apathy, perceived stigma, extroversion, subjective well‐being, resilience, hope, self‐esteem and social support as predictors of loneliness in PD and FSCD. The secondary aim of the study was to investigate the effects of age, gender and perceived stigma on loneliness in PD and FSCD. We hypothesized that older patients, male patients and patients with higher perceived stigma would feel lonelier.

## Methods

### Participants

Eighty‐two (150 initially contacted, 98 responded–65% response rate) people with PD, 63 (98 initially contacted, 66 responded–67% response rate) people with idiopathic FSCD and 50 (77 initially contacted, 50 responded–65% response rate) HCs were recruited from the patient lists of 3 collaborating consultant neurologists (PL, KB, and PJ) at the National Hospital of Neurology and Neurosurgery, Queen Square, London. All patients had been diagnosed as having idiopathic PD according to the criteria of the UK Brain Bank^41^ or FSCD (blepharospasm, cervical dystonia, Meige syndrome^30^). The HCs were recruited through social and community networks. HC participants were invited to participate if they had no chronic neurological, psychiatric or physical diseases and were over 40 years of age. All eligible participants were sent an invitation to take part in the study. The people who responded to the invitation to participate were contacted after a revision of the medical documentation. Any patients with dementia were excluded at the recruitement phase when examining their medical records and before sending out the questionnaire packs. Therefore, only non‐demented subjects whose ability to communicate was determined based on the available medical records and the telephone interview were allowed to participate in the study. These participants were sent a questionnaire pack. As suggested by the Ethics Committee who reviewed and approved the proposal, the return of the completed booklet acted as implied consent. Full ethical approval was granted by Health Research Authority and Health and Care Research Wales Ethics Committee. Full ethics approval for the project was obtained on February 2, 2018, application number 233401, REC Ref 18/LO/1368.

The sample size required to conduct the study was estimated based on a significance level of 0.05, a power of 0.80 and a medium‐high effect size based on previous research.^37^ A mean effect size of 0.35 was expected for this study; using power statistics the estimated minimum sample size was set to 57 per group. The minimum number of participants was recruited in each group, except for HCs, as recruitment had to be stopped due to the outbreak of the Covid‐19 pandemic.

### Measures

The questionnaire pack consisted of a cover sheet which required participants to divulge demographic and clinical information on age, gender, and disease duration. Table 1 displays the mean values and standard deviation of the mean on the questionnaires and scales used to assess the subjective and physical well‐being of the participants completed by each group. The following scales and questionnaires were used in the study (a detailed description of the measures is given in the Data S1): The UCLA Loneliness Scale (LS),^42^ The self‐reported stage of illness in PD,^43^ EQ‐5D,^44^ PDQ‐8^45^ in PD, The Non‐Motor Symptoms Scale (NMSS)^46^ in PD, The Hospital Anxiety and Depression scale (HADS),^47^ The Starkstein Apathy Scale (SAS)^48^ in PD, The Subjective Happiness Scale (SHS),^49^ The State Hope Scale (SHoS),^50^ The Stigma Scale,^51^ The Short Social Support Questionnaire (SSSQ),^37^ Life Orientation Test (LOT),^52^ Self‐Esteem Questionnaire (SEQ),^53^ and The Eysenck Personality Questionnaire Short Form (EPQ‐SF).^54^

**TABLE 1:** Demographic, clinical and measures of loneliness and well‐being in Parkinson's disease (PD), focal/segmental cervical dystonia (FSCD) and healthy controls (HC)

	PD	Dystonia	HC	ANOVA, *χ* ^2^	PD versus FSCD	PD versus HC	FSCD versus HC
Age (years)	68.16 ± 8.17	67.84 ± 10.27	58.76 ± 10.89	*F*(2,179) = 0.54	/	/	/
				*p* = 0.586			
				*η* ^2^ = 0.11			
Gender F:M	40:42	40:23	28:22	*χ* ^2^(2) = 25.36	/	/	/
				*p* = 0.28			
Disease duration (months)	80.04 ± 54.21	293.84 ± 151.87	/	/	*t*(135) = 29.51	/	/
					***p* = 0.001**		
					*d* = 1.89		
Loneliness scale, UCLA	38.63 ± 10.48	41.62 ± 13.24	36.75 ± 8.30	*F*(2,183) = 2.62	*t*(124) = 1.44	*t*(124) = 1.03	*t*(106) = 2.17
				***p* = 0.029**	*p* = 0.076	*p* = 0.153	***p* = 0.018**
				*η* ^2^ = 0.281	*d* = 0.25	*d* = 0.18	*d* = 0.42
Subjective happiness scale	5.01 ± 0.89	4.83 ± 1.13	5.60 ± 0.90	*F*(2,190) = 9.19	*t*(142) = 1.09	*t*(142) = −3.64	*t*(110) = −3.91
				***p <* 0.001**	*p* = 0.277	***p <* 0.001**	***p <* 0.001**
				*η* ^2^ = 0.088	*d* = 0.18	*d* = 0.66	*d* = 0.74
Brief resilience scale	3.45 ± 0.69	3.45 ± 0.86	3.86 ± 0.75	*F*(2,187) = 5.06	*t*(138) = 0.06	*t*(128) = −3.13	*t*(108) = −2.56
				***p =* 0.007**	*p* = 0.996	***p* = 0.002**	***p* = 0.006**
				*η* ^2^ = 0.051	*d* = 0.11	*d* = 0.56	*d* = 0.49
State hope scale: Trait hope agency subscale	21.94 ± 5.37	19.18 ± 4.39	22.73 ± 3.85	*F*(2,179) = 7.97	*t*(131) = 3.01	*t*(125) = −0.86	*t*(102) = −4.17
				***p <* 0.001**	***p* = 0.003**	*p* = 0.378	***p <* 0.001**
				*η* ^2^ = 0.082	*d* = 0.53	*d* = 0.16	*d* = 0.81
Life orientation test	14.51 ± 3.97	14.22 ± 5.21	18.40 ± 4.28	*F*(2,186) = 14.39	*t*(131) = 0.364	*t*(127) = −5.21	*t*(102) = −4.47
				***p <* 0.001**	*p* = 0.716	***p <* 0.001**	***p <* 0.001**
				*η* ^2^ = 0.134	*d* = 0.16	*d* = 0.94	*d* = 4.89
Self‐esteem questionnaire	21.39 ± 4.21	19.25 ± 5.19	18.22 ± 8.37	*F*(2,187) = 4.87	*t*(137) = 2.65	*t*(127) = 2.82	*t*(108) = 0.774
				***p =* 0.009**	***p* = 0.009**	***p* = 0.006**	*p* = 0.440
				*η* ^2^ = 0.049	*d* = 0.45	*d* = 0.51	*d* = 0.15
Stigma scale	7.57 ± 3.85	7.43 ± 4.24	/	/	*t*(139) = 2.66	/	/
					***p* = 0.009**		
					*d* = 0.32		
EQ‐5D‐Mobility	2.32 ± 1.09	1.98 ± 1.05	1.15 ± 0.35	*F*(2,187) = 23.04	*t*(140) = 1.84	*t*(127) = 7.21	*t*(107) = 5.19
				***p <* 0.001**	*p* = 0.069	***p <* 0.001**	***p <* 0.001**
				*η* ^2^ = 0.198	*d* = 1.08	*d* = 1.31	*d* = 1.01
EQ‐5D‐Pain/Discomfort	2.15 ± 0.89	2.54 ± 0.93	1.58 ± 0.57	*F*(2,187) = 17.34	*t*(140) = −2.53	*t*(127) = 3.91	*t*(107) = 6.19
				***p <* 0.001**	***p* = 0.012**	***p <* 0.001**	***p <* 0.001**
				*η* ^2^ = 0.156	*d* = 0.31	*d* = 0.71	*d* = 1.19
EPQ‐SF: Extraversion	6.22 ± 3.36	4.87 ± 3.34	7.47 ± 3.34	*F*(2,171) = 6.71	*t*(125) = 2.09	*t*(127) = −1.92	*t*(98) = −3.65
				***p =* 0.002**	***p* = 0.039**	*p =* 0.058	***p <* 0.001**
				*η* ^2^ = 0.073	*d* = 0.37	*d* = 0.35	*d* = 0.73
Hospital anxiety and depression score–anxiety subscale	7.33 ± 4.08	7.73 ± 4.49	5.46 ± 5.51	*F*(2,186) = 3.53	*t*(139) = −0.54	*t*(127) = 2.17	*t*(106) = 2.31
				***p =* 0.031**	*p* = 0.588	***p =* 0.031**	***p =* 0.023**
				*η* ^2^ = 0.037	*d* = 0.19	*d* = 0.39	*d* = 0.45
Hospital anxiety and depression score–depression subscale	5.96 ± 3.26	5.63 ± 3.62	2.98 ± 3.22	*F*(2,179) = 12.50	*t*(140) = 0.56	*t*(127) = 4.99	*t*(106) = 3.89
				***p <* 0.001**	*p* = 0.578	***p <* 0.001**	***p <* 0.001**
				*η* ^2^ = 0.118	*d* = 0.15	*d* = 0.91	*d* = 0.75
Hoehn & Yahr‐self rated	2.17 ± 1.27	/	/	/	/	/	/
PDQ‐8 total score	8.40 ± 5.99	/	/	/	/	/	/
Starkstein apathy scale: Cognitive aspects subscale	11.81 ± 4.93	/	/	/	/	/	/
Starkstein apathy scale: General apathy subscale	6.62 ± 4.90	/	/	/	/	/	/
Non‐motor symptoms scale	11.33 ± 11.28	/	/	/	/	/	/
Social support questionnaire: practical support quantity	4.28 ± 2.20	3.27 ± 2.43	/	/	*t*(128) = 2.33	/	/
					***p* = 0.021**		
					*d* = 0.43		
Social support questionnaire: practical support quality	5.49 ± 0.80	5.30 ± 1.18	/	/	*t*(125) = 1.01	/	/
					*p* = 0.318		
					*d* = 0.18		
Social support questionnaire: emotional support quantity	3.66 ± 2.43	3.04 ± 2.53	/	/	*t*(128) = 1.35	/	/
					*p* = 0.318		
					*d* = 0.24		
Social support questionnaire: emotional support quality	5.51 ± 0.84	5.28 ± 1.07	/	/	*t*(128) = 1.27	/	/
					*p* = 0.205		
					*d* = 0.23		

The values are presented as mean ± standard deviation of the mean. The significant *p*‐values are presented in bold.

Abbreviations: EQ‐5D, EuroQol 5 Dimensions; EPQ‐SF, Eysenck personality questionnaire short form; F, female; M, male; PDQ‐8, Parkinson's disease quality of life‐8 items.

### Statistical Analysis

IBM SPSS v29 for Mac was used for analysis. The significance level was set at 0.05. False discovery rate was used to correct for multiple comparisons.^55^ A *χ* ^2^‐test was performed to determine whether the proportion of genders was equal between groups. To assess the differences between patients with PD, FSCD and HC in loneliness a one‐way ANOVA was completed, with group as the between groups factor. In addition, a series of ANOVAs were conducted to assess possible differences in potential predictor variables of loneliness (see below). Independent groups’ two‐tailed *t*‐tests were used for planned post‐hoc analysis to further assess the differences between PD, FSCD, and HCs and to assess the differences in disease duration, stigma scale and social support questionnaire—the latter two completed only by people with PD and FSCD. A median cut‐off for age and for perceived stigma was used to perform a post‐hoc analysis of the effect of age and perceived stigma on loneliness in PD and dystonia using independent *t*‐tests. The effect of gender on loneliness was also analyzed using independent *t*‐tests.

In order to assess which variables predicted loneliness, multiple regression analyses were carried separately for PD and FSCD patients, with demographic and clinical measures [age, gender, disease duration], subjective well‐being/happiness (SHS), resilience (BRS), trait hope agency, life orientation (LOT), perceived stigma, mobility and pain/discomfort items from the EQ‐5D, extroversion from EPQ‐SF, anxiety (HADS), depression (HADS), practical and emotional social support quantity and quality (SSSQ), self‐esteem (SEQ), for both PD and FSCD and additionally for PD only quality of life (PDQ8), apathy (Starkstein apathy scale), non‐motor symptoms (NMSS), and self‐rated Hoehn and Yahr scale as predictors. The zero correlations between the variables for PD and FSCD are given in Data S2. The rate of missing values in the variables used in the regression analysis was low (less than 3%). Therefore, the missing values were not replaced, and the regression analysis was executed on the original variables.

## Results

### Differences in Clinical and Psychological Measures

The results of the detailed analysis are presented in Table 1. There was no statistically significant difference in age between groups, *p* = 0.586. On average, the participants with FSCD had a longer disease duration than those with PD, *p* = 0.001. The distribution of gender was not significantly different across the three groups, *p* = 0.281.

There was a significant difference in *loneliness* between groups *p* = 0.029. There was no significant difference in loneliness between people with PD and HCs, *p* = 0.153. Even though the people with FSCD reported higher loneliness levels than people with PD, this difference was not significant, *p* = 0.076. There was a significant difference in loneliness between people with FSCD and HC, *p* = 0.018, with the former reporting higher levels of loneliness.

### Predictors of Loneliness in PD and FSCD

The full regression models for both PD and FCSD are given in the Data S3. The regression model predicting loneliness in PD was significant *F*(23, 58) = 6.18, *p* < 0.001, adjusted *R* ^2^ = 0.71. Perceived stigma significantly predicted loneliness (*β* = 0.37, 95% CI [0.41, 1.59], *p* = 0.002), as did life orientation (*β* = −0.25, 95% CI [−1.17, −0.13], *p* = 0.017), non‐motor symptoms (*β* = 0.26, 95% CI [0.07, 0.41], *p* = 0.008) and the quantify of practical support subscale of the social support scale (*β* = −0.42, 95% CI [−9.31, −1.61], *p* = 0.007).

The regression model predicting loneliness in FSCD was also significant *F*(18, 44) = 6.02, *p* < 0.001, adjusted *R* ^2^ = 0.71. Perceived stigma was the only factor predicting loneliness in FSCD patients (*β* = 0.31, 95% CI [0.12, 1.80], *p* = 0.028).

### Effect of Gender, Age and Perceived Stigma on Loneliness in PD and FCSD

The results of the analysis are presented in Table 2.

**TABLE 2:** Effect of gender, age and stigma on loneliness as measured by the UCLA Loneliness Scale in Parkinson's disease (PD) and focal/segmental cervical dystonia (FSCD). A median cut‐off of 70 and 69 was used to analyze the effect of age on loneliness in PD and dystonia, respectively. A median cut‐off of 8 on the perceived stigma scale was used to evaluate the effect of low vs. high perceived stigma levels in PD and dystonia.

	Gender		
	Female	Male	*t*‐test
PD	42.00 ± 11.58	35.05 ± 9.04	*t*(75) = 2.96, ***p* = 0.004**, *d* = 0.68
FSCD	43.40 ± 14.02	38.74 ± 12.58	*t*(58) = 1.38, *p* = 0.198, *d* = 0.35

	Age		
	Younger	Older	*t*‐test
PD	36.00 ± 9.85	41.77 ± 11.58	*t*(73) = −2.33, ***p* = 0.023**, *d* = 0.55
FSCD	47.00 ± 13.47	37.55 ± 12.33	*t*(56) = 2.82, ***p* = 0.007**, *d* = 0.75

	Stigma		
	Less stigma	More stigma	*t*‐test
PD	33.63 ± 8.99	45.81 ± 8.89	*t*(76) = −5.91, ***p <* 0.001**, *d* = 1.36
FSCD	34.48 ± 11.37	50.38 ± 10.95	*t*(57) = −5.49, ***p <* 0.001**, *d* = 0.57

The significant *p*‐values are presented in bold.

Female patients with PD reported higher loneliness compared to males, *p* = 0.004. There was no significant effect of gender on loneliness in FSCD, *p* = 0.198.

A median cut‐off of 70 and 69 was used to analyze the effect of age on loneliness in PD and dystonia, respectively. Older patients with PD were more lonely than younger patients, *p* = 0.023. By contrast, younger patients with FSCD were lonelier than older patients, *p* = 0.007.

A median cut‐off of 8 on the perceived stigma scale was used to evaluate the effect of low vs. high perceived stigma levels in PD and dystonia. Both, PD, *p* < 0.001 and FSCD, *p* < 0.001 patients with higher experience of perceived stigma were also lonelier.

## Discussion

The main findings of the study were that loneliness was significantly higher in people with FSCD compared to HCs, but there was no difference in loneliness between PD and FSCD and PD and HCs. While perceived stigma significantly predicted loneliness in both PD and FSCD, non‐motor symptoms, lack of optimism and practical support additionally predicted loneliness in PD. Furthermore, patients with PD and FSCD with higher experience of perceived stigma were lonelier than those with lower levels of perceived stigma. Female and older patients with PD and younger patients with dystonia reported greater loneliness.

In the past couple of decades much has been published about the loneliness epidemic sweeping the modern Western world.^56^, ^57^ It is therefore informative rather than surprising that there was no significant difference in self‐reported loneliness between people with PD and HCs. Loneliness is a universal emotion which can be experienced by anyone.^58^ However, people with FSCD showed significantly higher levels of loneliness compared to HCs, but there was no difference in loneliness between PD and FSCD. Both disorders, PD and FSCD lead to motor difficulties making it challenging to participate in social activities. In addition, both conditions lead to difficulties in communication which can further enhance the feeling of loneliness.^21^ Depression and anxiety, common in both disorders can also exacerbate feelings of loneliness.^28^, ^33^, ^34^ In addition, family and caregivers might be heavily focused on managing the diseases sometimes unintentionally neglecting the emotional needs of the patients leading to loneliness.^59^, ^60^ Unlike female patients with FSCD, female patients with PD reported higher levels of loneliness. This finding of female people with PD showing higher levels of loneliness is in line with previous research, in which the gender differences increased as the disability severity increased.^61^, ^62^ Another explanation to this finding is that males are less likely to report loneliness than females.^63^ We found that older people with PD are lonelier, which can be explained by increase of mobility difficulties and social isolation, increase of cognitive decline in older patients.^61^ However, other studies have found that younger people show higher levels of loneliness than older people, which is in line with the finding in our study that younger patients with FSCD were lonelier than older patients.^63^ Disease duration was longer in FSCD due to disease onset at younger age, which might explain our findings. The absence of gender differences in loneliness in FSCD, suggests that both sexes report loneliness equally.

Looking at the predictors of loneliness; while perceived stigma predicted loneliness in both FSCD and PD, the non‐motor symptoms, and lack of practical support and absence of optimism predicted loneliness in people with PD. Perceived stigma is a devaluating, discriminant, and discomforting feeling caused by the negative perception of self and by others when the stigmatized person is perceived as different from the “norm.”^64^ In a recent study, perceived stigma was assessed using four different stigma scales in an online and an in‐person cohort of PD patients.^65^ Younger age and depression predicted perceived stigma in the online and in‐person samples, indicating the cross‐modality of the PDQ‐39 stigma subscale.^65^, ^66^ Anxiety predicted stigma perception in the online sample as measured by the PDQ‐39 stigma subscale and the Stigma Scale for Chronic Illness.^65^, ^67^ Similar findings were reported from an earlier study exploring the perceived stigma in PD using the PDQ‐39 stigma subscale, in which younger age in men and depression in both men and women predicted perceived stigma.^68^ Perceived stigma was a common significant predictor of loneliness in both people with PD and people with FSCD and this finding was expected as both movement disorders have commonly been associated with perceived stigma.^32^, ^69^ In addition, we have also found that loneliness was more pronounced in patients with PD and FSCD who scored higher on the stigma scale. Many qualitative studies have shown in‐depth accounts of how negative body image, shame, and embarrassment about the motor and non‐motor symptoms of both PD and FSCD have caused people with these conditions to hide from society and self‐isolate.^64^, ^70^ We had previously formulated a model of social avoidance in dystonia^71^, ^72^ in which a sense of disfigurement associated with FSCD can produce a negative body image and perceived stigma and then lead to social avoidance and isolation. Our current finding of perceived stigma as a predictor of loneliness in FSCD supports this model, which requires further validation in future studies by including some of the other relevant measures.

It has been suggested that sometimes the impact of non‐motor symptoms, which were found to significantly predict loneliness in PD, is worse than the impact of motor symptoms.^73^ For example, fatigue and mood disturbances can make it hard to engage, and dribbling can cause embarrassment, enhancing perceived stigma and in turn loneliness in people with PD.

Whereas the results show that perceived stigma and non‐motor symptoms have a negative impact on social connectedness, optimism and practical support both have more protective qualities, therefore it was not surprising that lack of optimism predicted loneliness in PD. Optimism is a personality dimension defined by the expectation of positive outcomes, life engagement, and a future orientation.^74^ Optimism is also related to motivation; optimistic people are motivated to exert effort socially.^75^ Optimism in PD has been positively associated with QoL and inversely related with the motor and non‐motor symptoms assessed using the UPDRS,^76^ suggesting that there is a relationship between optimism and the effects of PD that helps patients to remain more socially engaged.

The SSSQ^37^ assesses both the quality and quantity of support experienced by the patient. Low levels of practical social support also significantly predicted loneliness. Being supported in a way that satisfies the needs of the individual implies a level of awareness of the needs of the person with PD, by the person giving support. Social connections in PD such as “family” and “friends” are important constructs of life satisfaction as is “being heard” since it promotes feelings of acceptance and assurance,^77^ again promoting feelings of connectedness.

Further research could explore the effects of duration of disease as well as the neurodegenerative nature of PD versus the more static nature of FSCD. We did not assess the non‐motor symptoms of people with FSCD, which is one of the limitations of this study along with the small sample size, which could also be considered for any future research. Comparing other neurological disease groups such as essential tremor or multiple sclerosis, as comparative groups, could also shed light on more disease‐specific features which contribute to loneliness.

There are several limitations of the study. We collected the data by sending the questionnaires and scales to the patients by post. Therefore, we were not able to consider the objective motor status of the patients who participated in the study and used only the self‐reported stage of illness, which was nevertheless shown to correctly reflect the motor stage of PD.^43^ In addition, most of the scales used in the study have not yet been validated in PD and dystonia. However, there are no specific scales to measure loneliness in PD and dystonia, leaving us with the possibility of using scales validated in the general population. We used the usual significance level of 0.05. Due to the large number of variables and subscores it might have been more optimal to use a more conservative significance level of 0.01. However, the number of participants was planned based on the significance level of 0.05. In addition, we used false discovery rate to correct for multiple comparisons.

In conclusion, our study supports the notion that people with CND are lonelier than HCs. We found higher levels of loneliness in FSCD patients compared to HC. Perceived stigma was a significant predictor of loneliness in both, PD and FSCD patients. In addition, patients that felt more stigmatized also felt lonelier. Non‐motor symptoms, lack of optimism and practical support predicted loneliness in PD. Female patients with PD felt lonelier than male patients. In addition, older patients with PD and younger patients with FSCDS were lonelier. Further research is needed to understand the underlying intricacies of what contributes to loneliness in PD and FSCD as well as other movement disorders.

## Author Roles

(1) Research project: A. Conception, B. Organization, C. Execution; (2) Statistical Analysis: A. Design, B. Execution, C. Review and Critique; (3) Manuscript: A. Writing of the first draft, B. Review and Critique.

S.S.: 1A, 1B, 1C, 2C, 3A, 3B

D.G.: 2A, 2B, 2C, 3B

P.J.: 1C, 3B

K.B.: 1C, 3B

P.L.: 1C, 3B

M.J.: 1A, 1B, 1C, 2A, 2C, 3B

## Disclosures

**Ethical Compliance Statement:** Full ethical approval was granted by Health Research Authority and Health and Care Research Wales Ethics Committee (number 233401, REC Ref 18/LO/1368). As suggested by the Ethics Committee who reviewed and approved the proposal, the return of the completed booklet acted as implied consent to participate in the study. We confirm that we have read the Journal's position on issues involved in ethical publication and affirm that this work is consistent with those guidelines.

**Funding Sources and Conflicts of Interest:** DG, was partially supported by the Slovenian Research and Innovation Agency (ARIS) project programme P2‐0209 Artificial intelligence and intelligent systems. The authors declare that there are no conflicts of interest relevant to this work.

**Financial Disclosures for the Previous 12 Months:** The authors declare that there are no additional disclosures to report.

## Supporting information


**Data S1.** Detailed description of the scales and questionnaires.

**Data S2.** Zero correlations between the variables in the regression model for Parkinson's disease and focal/segmental craniocervical dystonia.

**Data S3.** Multiple regression models for Parkinson's disease and focal/segmental craniocervical dystonia.

## Data Availability

The data that support the findings of this study are available from the corresponding author upon reasonable request.
