# Supplementary material for: Predictors of Loneliness in Parkinson's Disease and Craniocervical Dystonia
Source: Mov Disord Clin Pract. 2025 Apr 29;12(9):1302–12. doi: 10.1002/mdc3.70098 (PMC12481432; doi:10.1002/mdc3.70098)
Supplement: Supplementary file 1 — Data S1. Detailed description of the scales and questionnaires. [file MDC3-12-1302-s003.docx]

**Supplementary Material 1: Detailed description of the scales and questionnaires**

*The UCLA Loneliness Scale (LS)*^1^ is a 20-item scale of which 9 are reverse scored aimed at measuring levels of loneliness. It asks questions such as, “How often do you feel alone?” Respondents are asked to respond on a 4-point scale where 1= Never and 4 = Always. Higher scores indicate higher levels of loneliness.

*The self-reported stage of illness in PD*^2^ is a 5-point scale to assess stage of illness in PD. In the modified version of the Hoehn & Yahr Scale^3^ for self-report used in this study, patients are asked about the presence of various symptoms, such as tremor, balance and gait problems, using a 5-point scale. Patients are also required to reflect on levels of aid they require. 0=I have no symptoms or slowness and 5=I am in a wheelchair (or bed) unless if I have help.

*EQ-5D*^4^ measures health using five levels of severity which are coded on a 5-point scale: 1=no problem, 2=slight, 3=moderate, 4=severe and 5=unable. These levels are scored across five dimensions: mobility, self-care, usual activities, pain and discomfort and anxiety and depression.

*PDQ-8*^5^ is a shortened version of the PDQ-39^6^. It is an 8-item scale that assesses quality of life in people with PD and is marked using a 5-point scale where 0=never, 1=occasionally, 2=sometimes, 3=often and 4=always. Each item relates to a different dimension: mobility, activities of daily living, emotional wellbeing, stigma, social support, cognition, communication and bodily discomfort.

*The Non-Motor Symptoms Scale (NMSS)*^7^ is a 30-item scale that assess the number of non-motor symptoms the person with PD is experiencing. It asks patients if they have experienced dribbling of saliva during the daytime, or feeling ‘sad’, ‘low’ or ‘blue’ in the past month. Patients are asked to respond ‘yes’ or ‘no” to each item. The total score is the number of items responded ‘yes”.

*The Hospital Anxiety and Depression scale (HADS)*^8^ is a 14-item scale that contains two subscales, one for anxiety one for depression with a possible overall score of 21 for each sub-scale. For anxiety or depression subscales, scores of 0-7 have been deemed to be within the normal range, 8-10 is borderline and scores of 11 and above suggest ‘caseness’.

*The Starkstein Apathy Scale (SAS)^9^ is* a 14-item scale that measures cognitive and behavioural aspects of apathy. These are graded on a 4-point scale where 0= a lot and 3 = not at all. Higher scores indicate higher levels of apathy.

*The Subjective Happiness Scale (SHS)*^10^ is a four item scale with statements such as “in general I consider myself” and respondents are asked to circle a number between 1-7 where 1 signifies “not a very happy person” and 7 is “a very happy person.” Higher scores indicate higher levels of happiness.

*The State Hope Scale (SHoS)^11^* is a 12 item questionnaire on which respondents are asked to rate 4 items which assess the respondents approach to pathways (Trait Hope Pathways) to achieving goals such as “There are lots of ways around any problem,” and 4 questions which assess the individual’s agency (Trait Hope Agency) in achieving goals such as, “I energetically pursue my goals.” 4 items are filler items. Higher scores indicate higher levels of search and agency.

*The Stigma Scale* ^12^. Sigma was assessed using the modified stigma scale, a 6-item scale whereby respondents indicate to what extent their illness has affected how they perceive themselves in relation to others and feel stigmatized by others. Answers are rated using a 4-point scale where 0=not at all and 3=definitely.

*The Short Social Support Questionnaire (SSSQ)*^13^ measures the quantity and quality (satisfaction with) of two elements of social support: practical and emotional support. Respondents are asked to mark down the initials of the friends or relatives who help them in each of these domains and are then asked to rate their levels of satisfaction for each dimension from 1-6 where 1=very dissatisfied and 6=very satisfied. Higher scores indicate higher levels of perceived social support.

*Life Orientation Test (LOT)^14^* is a 10-item scale that measures levels of optimism. 3 questions are positively worded such as, “In uncertain times I usually expect the best”, 3 items are negatively worded and reverse scored, such as, “If something can go wrong for me it will,” and there are 4 filler questions. Scores ranging from 0-13 suggest low levels of optimism, 14-18 suggest moderate levels of optimism and scores ranging from 19-24 suggest high levels of optimism.

*Self-Esteem Questionnaire (SEQ)^15^* is a 10-item questionnaire of which 5-items are positively worded, such as, “I take a positive attitude towards myself.” 5 items are negatively worded and reverse scored such as, “At times I think I am no good at all.” All items are scored on a 4-point Likert scale whereby 0 = strongly disagree and 3=strongly agree. Scores ranging from 0-15 suggest low levels of self-esteem, 15-25 suggest normal levels of self-esteem and 25-30 suggest high levels of self-esteem.

*The Eysenck Personality Questionnaire Short Form (EPQ-SF)*^16^ is a 48-items questionnaire. Each item is a statement that requires the respondent to respond to questions with either yes or no. For example, “Does your mood go up and down?” or “Are you a worrier?” The EPQ-SF measures 4 personality traits whereby higher scores show higher levels of trait: 1. Psychoticism; characterised by tough mindedness. 2. Extraversion; characterised by levels of sociability, 3. Neuroticism; Characterised by levels of emotionality and anxiety, 4. Lie; characterised by levels of socially undesirable behaviours.

References:

1. Russell D, Peplau LA, Cutrona CE. The revised UCLA Loneliness Scale: concurrent and discriminant validity evidence. Journal of personality and social psychology 1980;39(3):472.

2. Schrag A, Jahanshahi M, Quinn N. How does Parkinson's disease affect quality of life? A comparison with quality of life in the general population. Mov Disord 2000;15(6):1112-1118.

3. Hoehn MM, Yahr MD. Parkinsonism: onset, progression and mortality. Neurology 1967;17(5):427-442.

4. Herdman M, Gudex C, Lloyd A, et al. Development and preliminary testing of the new five-level version of EQ-5D (EQ-5D-5L). Quality of life research 2011;20(10):1727-1736.

5. Jenkinson C, Fitzpatrick R, Peto V, Greenhall R, Hyman N. The PDQ-8: development and validation of a short-form Parkinson's disease questionnaire. Psychology and Health 1997;12(6):805-814.

6. Peto V, Jenkinson C, Fitzpatrick R, Greenhall R. The development and validation of a short measure of functioning and well being for individuals with Parkinson's disease. Quality of life research 1995;4(3):241-248.

7. Chaudhuri KR, Martinez‐Martin P, Brown RG, et al. The metric properties of a novel non‐motor symptoms scale for Parkinson's disease: results from an international pilot study. Movement disorders 2007;22(13):1901-1911.

8. Zigmond AS, Snaith RP. The hospital anxiety and depression scale. Acta Psychiatr Scand 1983;67(6):361-370.

9. Starkstein SE, Mayberg HS, Preziosi TJ, Andrezejewski P, Leiguarda R, Robinson RG. Reliability, validity, and clinical correlates of apathy in Parkinson's disease. J Neuropsychiatry Clin Neurosci 1992;4(2):134-139.

10. Lyubomirsky S, Lepper HS. A measure of subjective happiness: Preliminary reliability and construct validation. Social indicators research 1999(2):137-155.

11. Snyder CR, Sympson SC, Ybasco FC, Borders TF, Babyak MA, Higgins RL. Development and validation of the State Hope Scale. J Pers Soc Psychol 1996;70(2):321-335.

12. MacDonald L, Anderson H. Stigma in patients with rectal cancer: a community study. Journal of Epidemiology & Community Health 1984;38(4):284-290.

13. Jahanshahi M, Marsden CD. Personality in torticollis: a controlled study. Psychological medicine 1988;18(2):375-387.

14. Scheier MF, Carver CS. Optimism, coping, and health: assessment and implications of generalized outcome expectancies. Health psychology 1985;4(3):219.

15. Rosenberg M. Rosenberg self-esteem scale (SES). Society and the adolescent self-image 1965.

16. Eysenck SB, Eysenck HJ, Barrett P. A revised version of the psychoticism scale. Personality and individual differences 1985;6(1):21-29.
